# Supplementary material for: Stimulus uncertainty and relative reward rates determine adaptive responding in perceptual decision-making
Source: PLoS Comput Biol. 2025 May 27;21(5):e1012636. doi: 10.1371/journal.pcbi.1012636 (PMC12143545; doi:10.1371/journal.pcbi.1012636)

Supplemental Figure 6

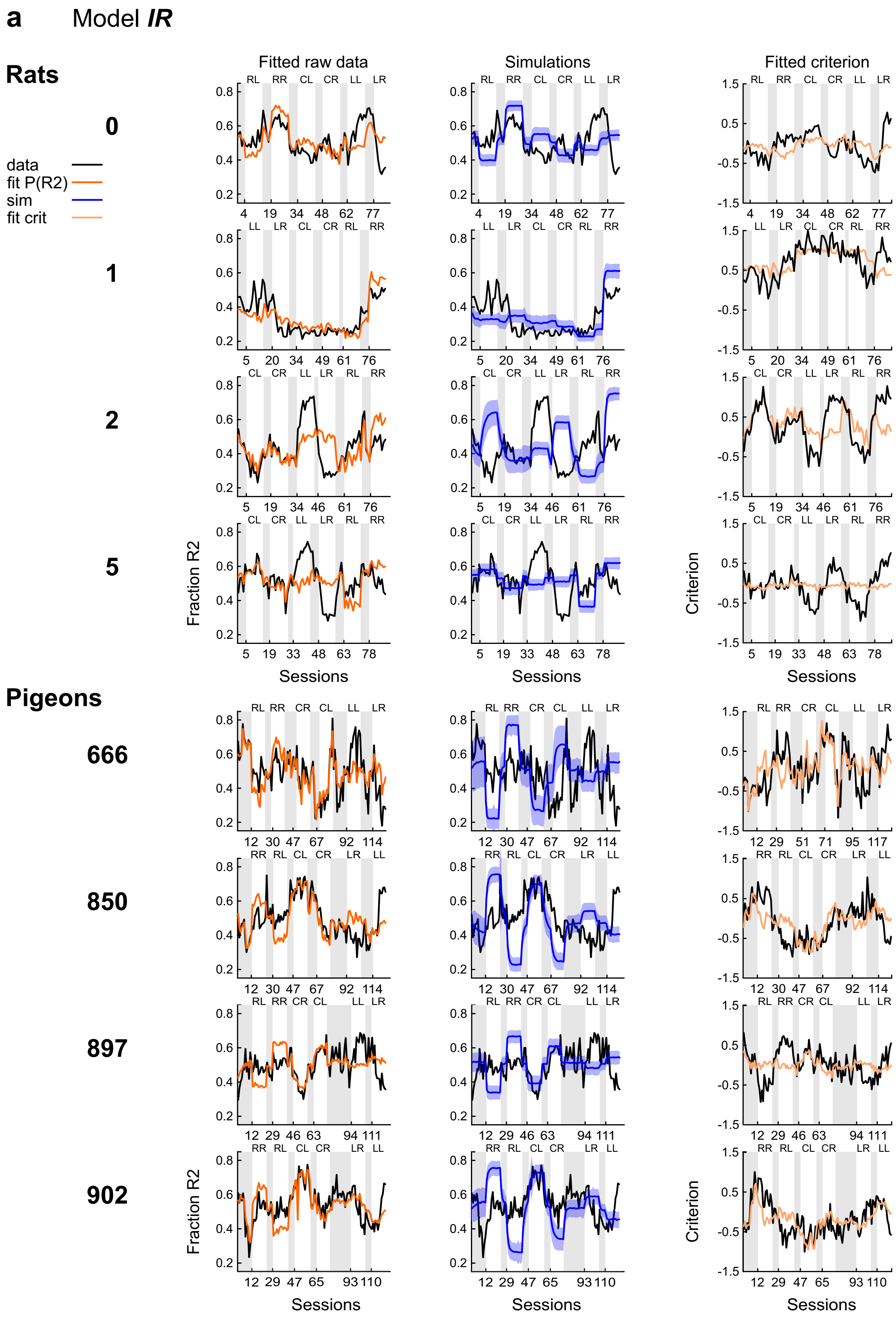

Supplemental Figure 6

b Model ***IR-SLR***

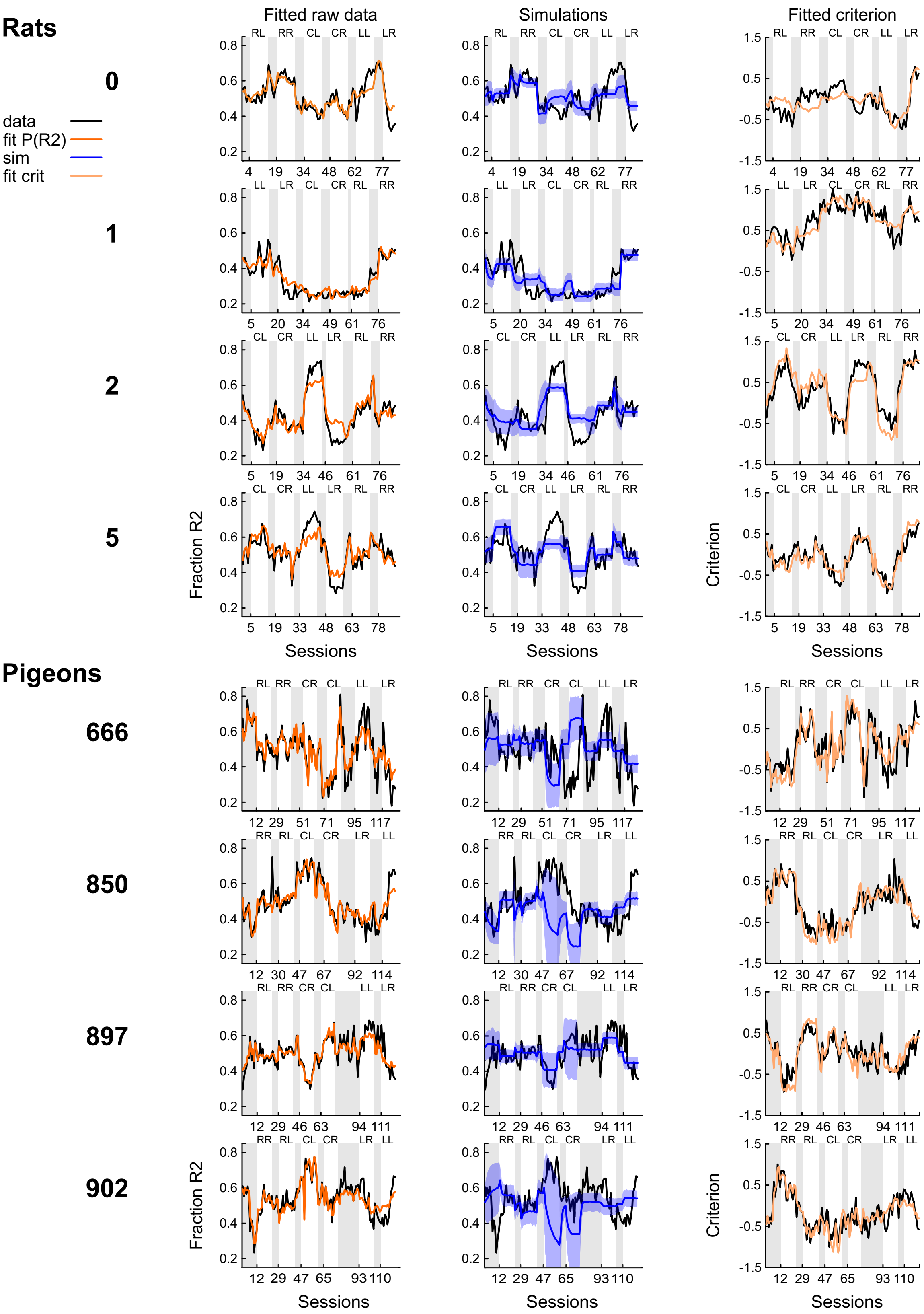

Supplemental Figure 6

c Model ***IR-RD***

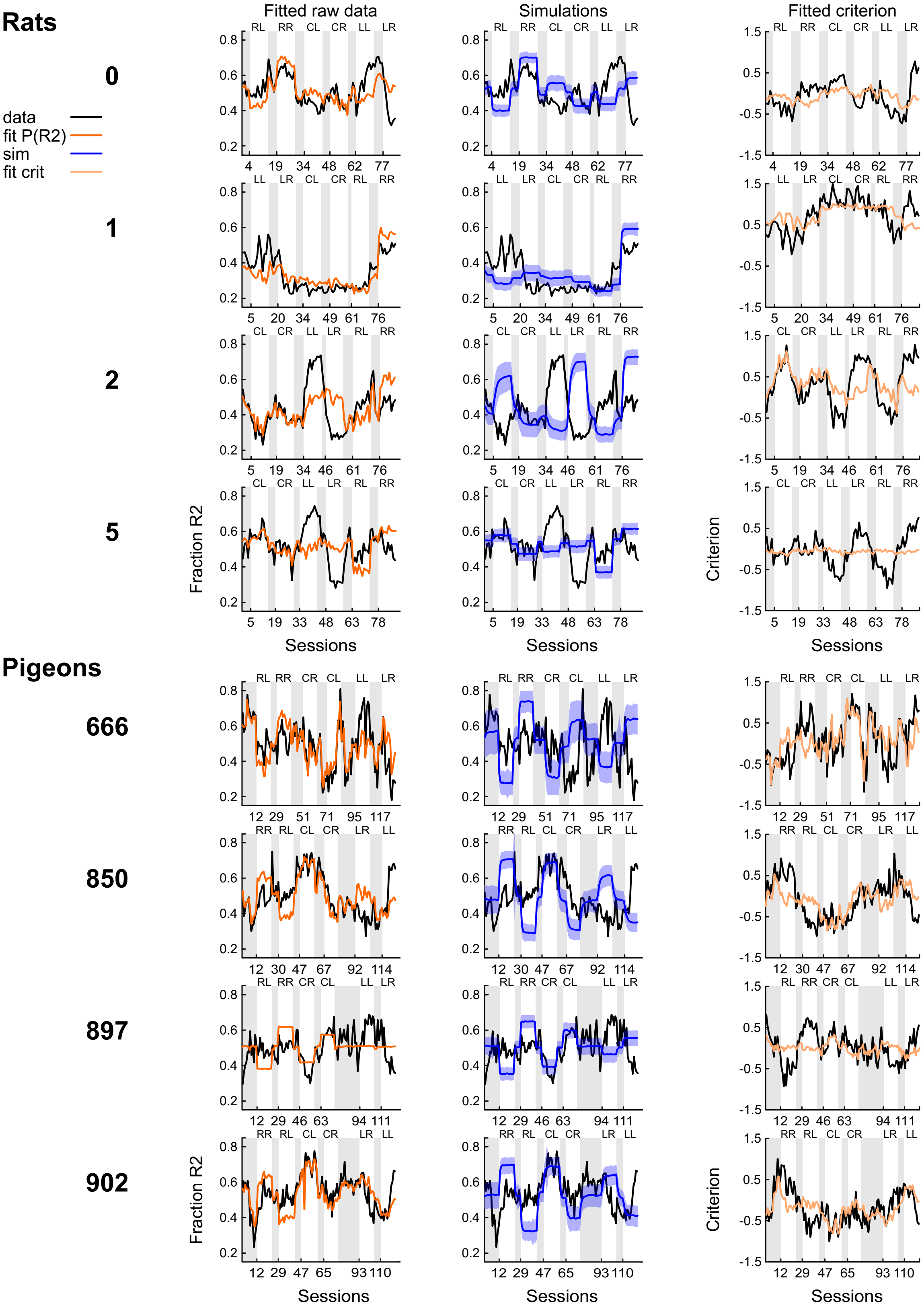

Supplemental Figure 6

d Model ***IR-SLR-RD***

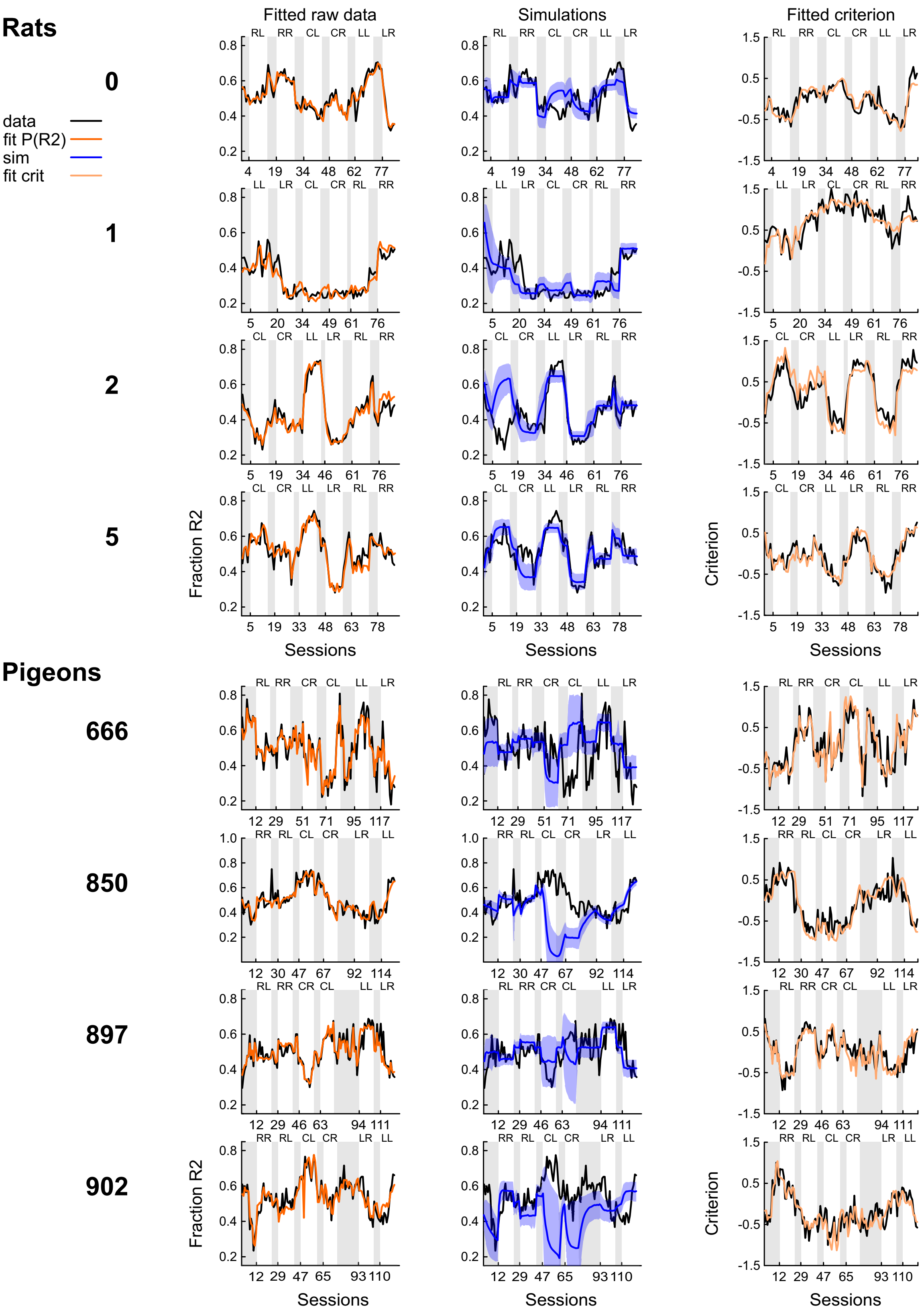

Supplemental Figure 6

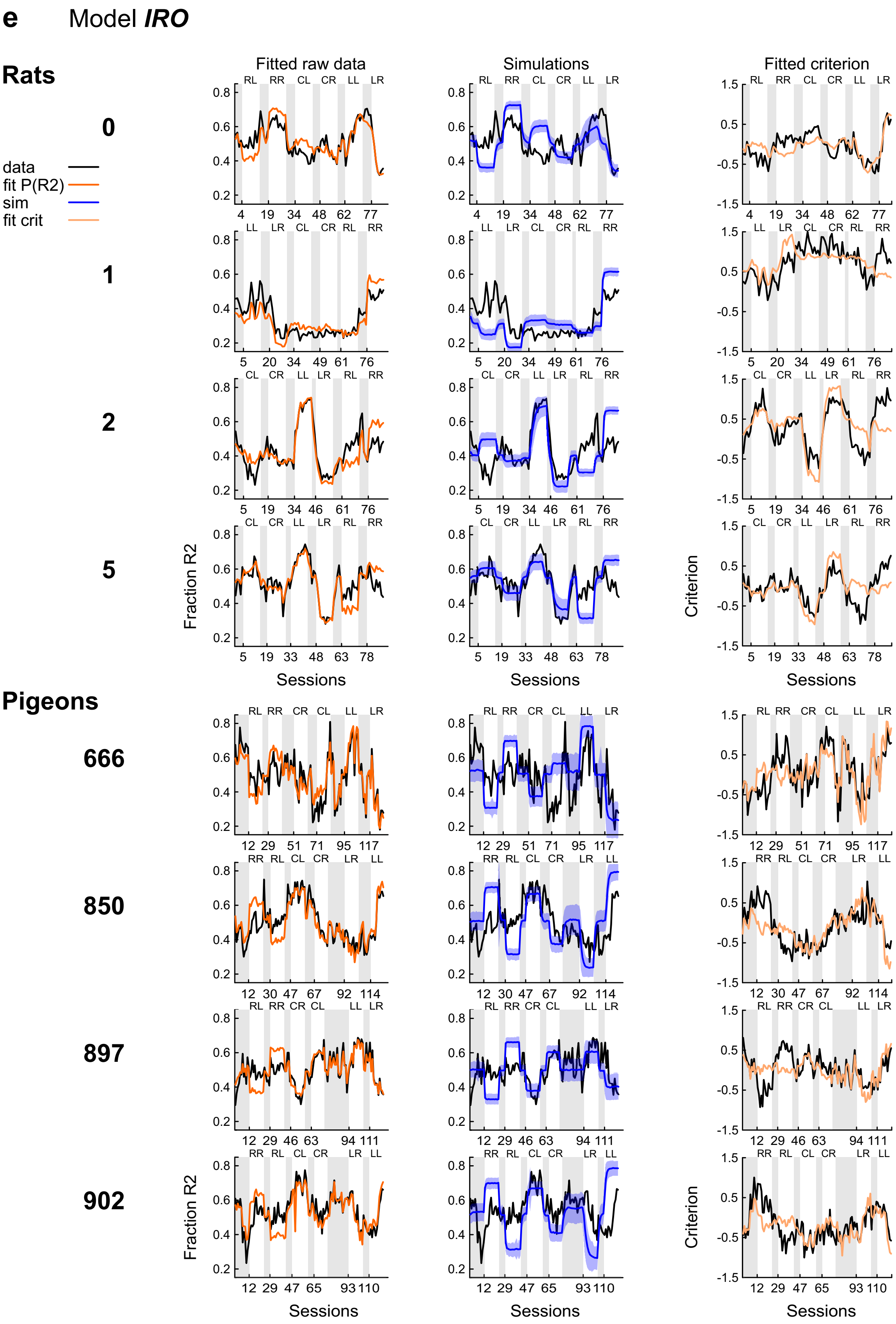

Supplemental Figure 6

f Model ***IR&RO***

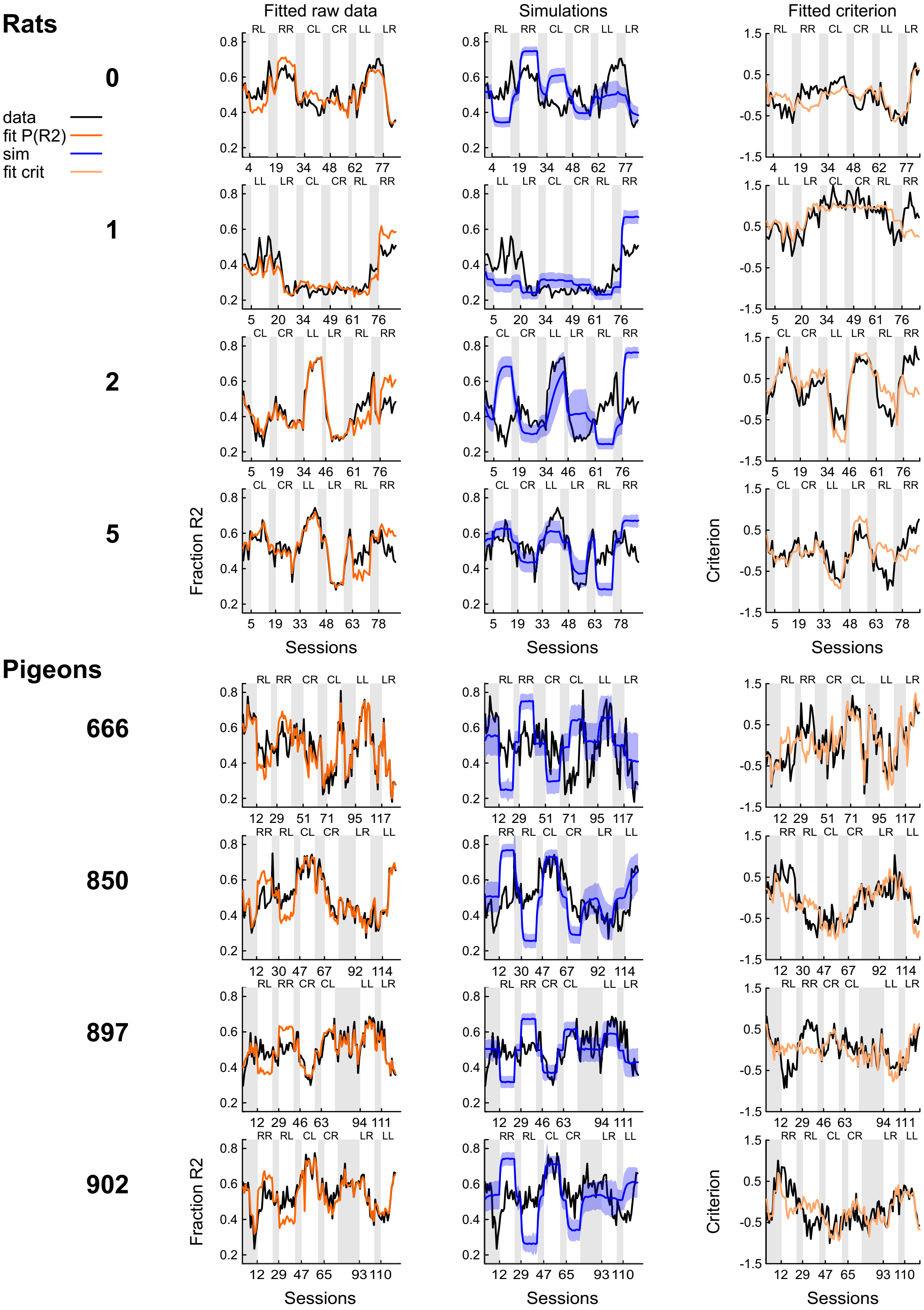

Supplemental Figure 6

g Model ***IR-SLR(red)***

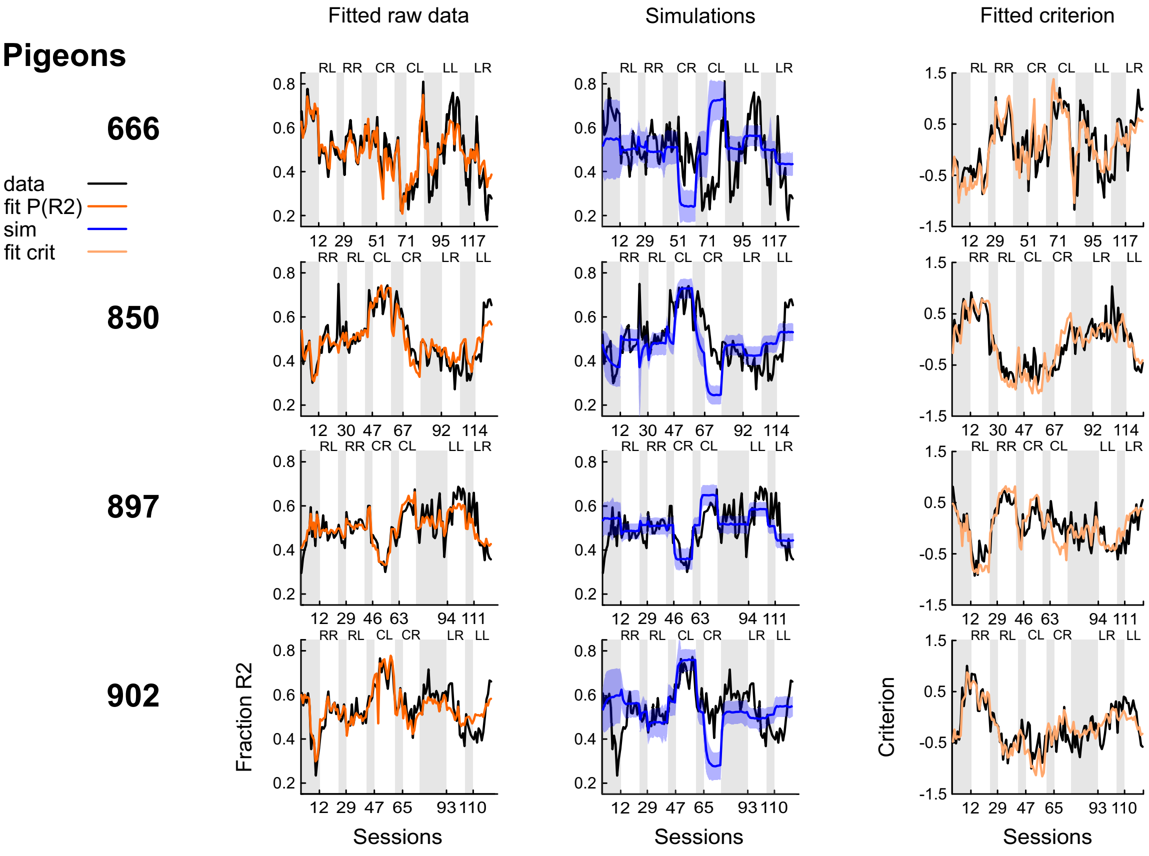

Supplemental Figure 6

h Model ***IR-SLR(red)-RD***

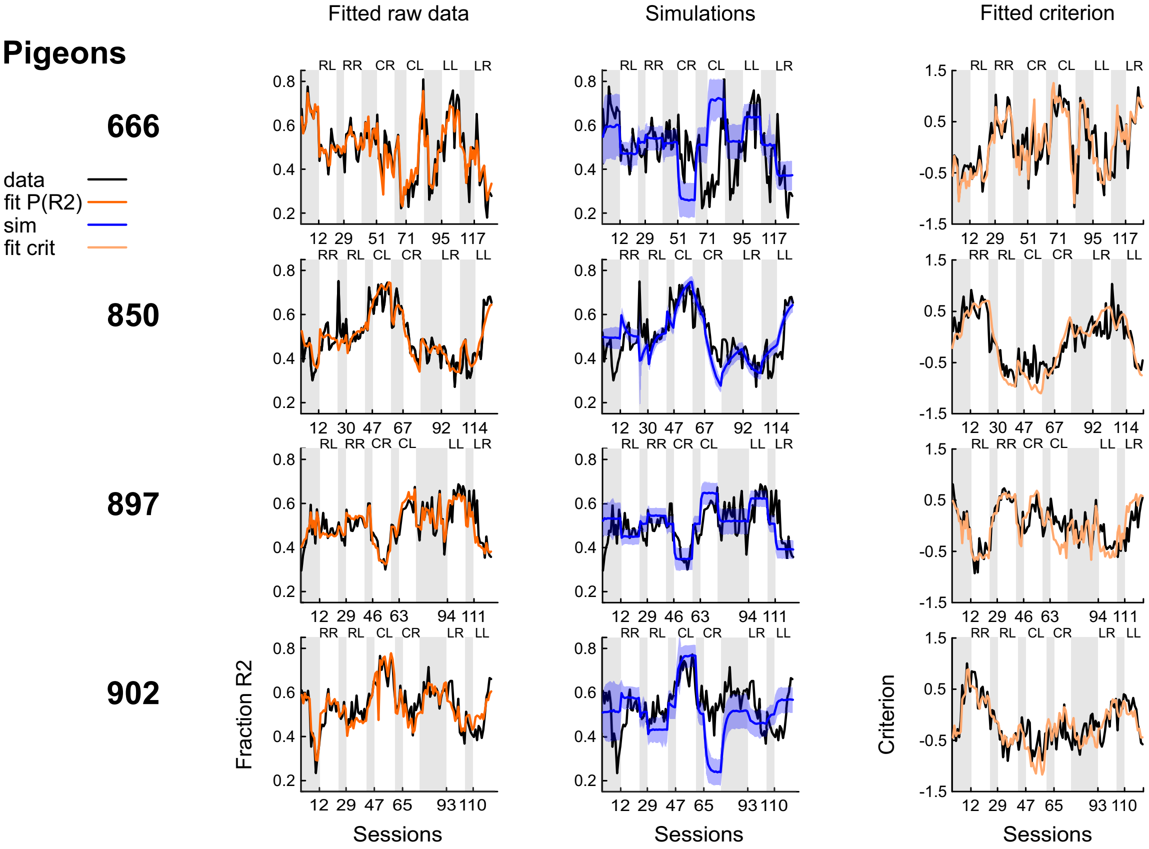

Supplement: S6 Fig — Individual fits (visualized as P(R2) and criterion) and simulations of different model versions considered in the main body of the manuscript for all rats and pigeons. a. IR model. b. IR-SLR model. c. IR-RD model. d. IR-SLR-RD model. e. IRO model. f. IR&RO model. g. IR-SLR(red). This model version features only two (instead of five) learning rates and is applied to pigeons only. h. IR-SLR(red)-RD, pigeons only, as in g. (PDF) [file pcbi.1012636.s006.pdf]
