# Supplementary material for: Stimulus uncertainty and relative reward rates determine adaptive responding in perceptual decision-making
Source: PLoS Comput Biol. 2025 May 27;21(5):e1012636. doi: 10.1371/journal.pcbi.1012636 (PMC12143545; doi:10.1371/journal.pcbi.1012636)
Supplement: Attachment — Submitted filename: Response to Reviewers v2.docx [file pcbi.1012636.s009.docx]

Response to Reviewers

Dear editors, dear reviewers,

thank you very much for providing helpful and detailed comments on our manuscript. We worked hard to address all issues and hope that the revised manuscript will find your approval.

Please find our point-by-point responses below.

# Editors

[…] both reviewers raised concerns about the clarity of the experimental design, the goals of the study, and its connections to existing literature (e.g., studies on matching experiments). Additionally, several statements in the Results section need to be supported with appropriate statistical tests. To strengthen the connection to previous literature, it would be helpful to include a discussion and references to studies examining the effects of global reward rate (e.g., Wittmann et al., Nature Communications, 2020) and reward uncertainty (Woo et al., Cognitive, Affective, & Behavioral Neuroscience, 2023) on learning and decision making. Lastly, please ensure precise language when discussing insight your study provides into the mechanisms underlying perceptual decision-making.

**Response**

We revised the manuscript in the following ways:

1) Abstract and Introduction were overhauled to more clearly state the aims of the study.
2) The experimental design is now described in detail in the main text. In addition, the new Figure 2 graphically illustrates the construction of the experimental conditions, and Table 3 (in Methods) supports this with numerical details.
3) The revised Results section includes both descriptive statistics and inferential statistical analyses to support our claims.
4) The connection to existing literature has been significantly strengthened: the revised Discussion section includes three additional subsections on issues raised by editors and reviewers, specifically: reward maximization / optimality, global reward rate and reward uncertainty, and a comparison with alternative models.

Also, we have rephrased many sentences to ensure precision and improve readability.

# Reviewer #1

Cuesta-Ferrer et al. investigate decision-making and its main determinants in pigeons and rats using a perceptual decision-making task (PDM). The authors manipulate reward probabilities, stimulus presentation probabilities, and discrimination difficulty, employing detection theory-based models to explain the behavior. They demonstrate that obtained rewards, rather than reward omissions, drive adaptation in behavior in response to changes in contingencies. While the manuscript addresses an important question, reliance on supplementary information for crucial experiment details and the lack of a comprehensive statistical report at many points hindered my ability to fully appreciate the significance of the findings and the motivation behind the study.

Major comments:

1) The topic of the manuscript has been widely studied in the field and will continue to be explored in the future. However, the introduction section of the paper does not adequately reflect this. Specifically, there should be a more thorough literature review on the models that utilize signal detection theory and confidence to explain adaptive behavior. Additionally, the paper should clarify the gaps in the existing literature that the authors aim to address, as the motivation for the study is currently unclear.

**Response**

Thank you very much for taking the time to read our manuscript and provide valuable comments. We apologize for not being clear about our motivation. Accordingly, we have rewritten the abstract and introduction, which has also been expanded to cover criterion variability, gaps in the literature, SDT-based models (also see the discussion section “Alternative models”), and we have stated our aims more clearly.

**Reviewer #1**

2) The abstract starts with "In an ever-changing environment, animals must learn to be flexible." This suggests that learning and reversals in learning are necessary. However, based on the methods section, all tests occurred after the animals became fully familiar with the task, and there was no reversal in the S-R map implemented.

**Response**

We agree that this sentence is misleading. In fact, all experimental testing (i.e., S-R-O contingency changes) occurred after the animals had learned the basic PDM task, including the basic S-R mapping (low pitch -> R1, high pitch -> R2). The repeated contingency changes during testing required the animals to flexibly adapt responding, which is what we refer to as “criterion learning” in the manuscript.

To avoid confusion, we implemented two changes:

a) We modified the sentence which now reads: “[…] animals must select actions based on sensory input and the expected positive and negative consequences.”

b) We speak more precisely of “criterion learning” when referring to adaptive behavior induced by contingency changes, and we discuss the learning of the initial S-R mapping (Discussion).

Regarding S-R remapping (reversal learning), please see our response to comment 5 below.

**Reviewer #1**

3) What is the neuroscience intuition behind changing the criteria thresholds in Fig 1, particularly in instances where S1 had an R2 response, and S2 had an R1 response? Additionally, the choice of a rewarding option relies on both perceptual acuity and an understanding of the rules. Consequently, moving the criterion line will attribute the reward solely to perception, which can lead to issues with reward assignment.

**Response**

This comment requires more detailed treatment.

First, it is important to note that SDT’s criterion is very different from the threshold concept used in classical psychophysics. In SDT, there is no such threshold, only internal noise to which a signal may or may not be added, resulting in a particular value x on the decision axis. The observer is assumed to adopt a decision criterion, then the decision rule is: if x>c, emit R2, if x<c, emit R1 (Green & Swets, 1988).
SDT separates the perceptual judgment into a sensory component and a decision component. We model the former with stimulus distributions whose locations are fixed throughout testing (an assumption supported by the observation that hit and false alarm rates change very little over months of testing; Figures 3d and S7b). The criterion is free to change at a trial-by-trial level, and it is precisely the question of how observers adopt (or “learn”) a particular criterion value that is the focus of our research.

Second, what is the “neuroscience intuition” behind criterion changes? While our goal here was not to develop a biologically plausible model, there are neural mechanisms that could implement criterion shifts.
In SDT, criterion shifts are conceptualized as purely decisional, so we would expect stimulus representations not to change when criterion shifts occur, which is supported by observations in monkey visual cortex (Luo & Maunsell, Neuron 86: 1182) and rat auditory cortex (Stoilova et al., J Neurophysiol 124: 1056).
Changes in criterion produce response bias (these measures are monotonically related). Bari et al. (Neuron 109: 922) reported that medial PFC neurons persistently represent the animal’s current response bias in a two-choice task with blockwise changing reward probabilities. This persistent representation of the criterion could, for example, lead to differential pre-activation of motor programs (Dorris & Munoz, J Neurosci 18: 7015) or representations of action target locations (Sugrue et al., Science 304: 1782). In the dorsomedial striatum, optogenetic activation of D1-/D2-expressing neurons produces a bias for contra- and ipsilateral responses, respectively (Tai et al., Nat Neurosci 15: 1281).
That said, neuronal representations of criterion are certainly more complex than just changes in firing rate. For example, in monkey lateral PFC, criterion changes affect not only firing rates but also the pattern of pairwise correlations and spiking regularity (Luo & Maunsell, Neuron 97: 1382).
The neuroscience intuition behind S1->R2 and S2->R1 could e.g. be a reduction of dopaminergic activity, i.e., a negative prediction error (Nomoto et al., J Neurosci 30: 10692). However, our model does not learn on non-rewarded trials, since our results suggest little to no influence of reward omissions after incorrect responses (Figure 5c).

Third, regarding the reviewer’s claim “moving the criterion line will attribute the reward solely to perception, which can lead to issues with reward assignment”.
If we understand the reviewer correctly, this point is about a credit assignment problem, i.e., that a reward will be attributed to a stimulus rather than to the response that produced it. This question seems somehow unrelated to our model architecture but more related to learning the stimulus-response mapping in the first place (see our response to comment 5). In our model, the observer has full knowledge of the stimulus distributions and the decision rule, just as in standard SDT. Shifting the criterion after a reward makes the rewarded response more likely to occur, but whether or not it occurs is always determined by a comparison of the current percept x with the current criterion value.
If the subject emits R1 after S2, this simply means that the current value of x is on the wrong side of the current criterion, and there will be no reward. In the IR-based models, the criterion does not change. In the IRO and the IR&RO models (rejected), the criterion changes so that the unrewarded response becomes less likely in the future.

**Reviewer #1**

4) The equations in Fig. 1b do not have a parameter indicating what the stimulus was. This is probably a notation issue, though.

**Response**

This is not a notation issue. The SDT decision rule (if x>c, emit R2; if x<c, emit R1) takes the percept (x) into account. The three initially considered models do not, but update according only on the basis of the outcome, so no stimulus parameter is necessary. The stimulus parameter however is introduced in the extended “SLR” versions of the models.

In the revised manuscript, we explain the SDT decision rule in the introduction with reference to Figure 1a. To more clearly differentiate the later models from the initial ones, we now additionally present the model equations in Figure 6a (also see Figure 1b).

**Reviewer #1**

5) How do the authors think the model works if there was a reversal in rule (S-R mapping)?

**Response**

Thank you for prompting us to consider this issue.

Our model does not include a mechanism to learn the initial stimulus-response mapping but only deals with adaptive choice behavior once that mapping has been learned. In other words, the S-R mapping is hard-coded in the model. There is of course no question that animals would eventually learn the reversal (in unpublished experiments with different auditory stimuli, we found it takes rats between 1 and 5 sessions to achieve >80% correct after S-R reversal was introduced).

In essence, the important question of reversal learning relates to that of how animals learn a two-stimulus two-response conditional discrimination task in the first place, and the same mechanism could be deployed to explain S-R reversal learning (see e.g., Bathellier et al., PNAS 110: 19950, for reinforcement-learning based model of task acquisition which however does not deal with stimulus uncertainty). While this was not the goal of our experiment, it would certainly be worthwhile to extend the model in this way in the future, relating to recent attempts to characterize initial learning (Roy et al., Neuron 109: 597; Daniels & Balsam, Animal Cognition 26: 1217).

We have addressed this issue in the discussion, section “Limitations and future directions”.

**Reviewer #1**

6) There are several important details, such as reward probabilities, that are referred to as supplementary results, but they should be clearly written in the main text rather than supplementary information.

**Response**

Thank you for this suggestion. We have merged Figure 1d and Figure S1.1 (see Figure 2 of the revised manuscript) and added a table (Table 1 of the revised ms) so that all important details are now included in the main text.

**Reviewer #1**

7) The experiment description for pigeons could use some revision to enhance comprehension. Figure 6.a and 6.b are too abstract, making it difficult to understand the experiment based on the description in the methods section.

**Response**

Thank you for pointing this out. We have completely overhauled Figures 6a and 6b (now Figures 7ab) to make them more understandable, and streamlined the experiment description for pigeons and rats (see new Figure 3ab). Also, we have added a brief description of the task to the figure legend.

**Reviewer #1**

8) There are many instances of statements in the results section that require a report of statistics, but nothing is reported (e.g., 4.3, 4.4, and 4.5).

**Response**

We now provide both descriptive and inferential statistics throughout the results section of the revised manuscript, including but not limited to the three sections 4.3, 4.4, and 4.5.

Again, thank you very much for your detailed comments which have helped us tremendously to improve the overall quality and intelligibility of our manuscript.

# Reviewer #2

This paper investigated decision making in rats and pigeons under different task contingencies that were determined based on reward probabilities, stimulus probabilities, and stimulus discriminability. Crucially, authors took account of trial by trial adjustments of decision thresholds under the signal detection theory framework. Authors tested three different models: reward, reward omission, and both. They find that the integration of the rewards considering stimulus difficulty and reward difference metrics (i.e., relative difference) best accounts for their data. Finally, the authors find that the performance of rats and pigeons were comparable in terms of model fits and that these animals nearly optimized their decisions (reward maximization). I find the paper very interesting and well-written. I have minor comments.

1- The paper seems to have overlooked a large set of literature that directly relates to the current work. I think the inclusion of these studies (primarily by the Balci group) to the paper is necessary given their direct relevance not only in terms of the results but also the research questions and the theoretical approach (e.g., statistical decision theory considering stimulus uncertainty, optimality).

- Balci, F., Freestone, D., & Gallistel, C. R. (2009). Risk assessment in man and mouse. Proceedings of the National Academy of Sciences of the United States of America, 106(7), 2459–2463. https://doi.org/10.1073/pnas.0812709106

- Tosun, T., Gür, E., & Balcı, F. (2016). Mice plan decision strategies based on previously learned time intervals, locations, and probabilities. Proceedings of the National Academy of Sciences of the United States of America, 113(3), 787–792. https://doi.org/10.1073/pnas.1518316113

- Akdoğan, B., & Balcı, F. (2016). Stimulus probability effects on temporal bisection performance of mice (Mus musculus). Animal cognition, 19(1), 15–30. https://doi.org/10.1007/s10071-015-0909-6

- Gür, E., Duyan, Y. A., & Balcı, F. (2019). Probabilistic Information Modulates the Timed Response Inhibition Deficit in Aging Mice. Frontiers in behavioral neuroscience, 13, 196. https://doi.org/10.3389/fnbeh.2019.00196

This is not a comprehensive list. I suggest the authors to look into these work.

**Response**

Thank you very much for these references. We were indeed aware of the work by the Balci lab. We have taken up the suggestion and have added a section which discusses our results in the context of reward maximization / optimality and model-based learning (as in Tosun et al.; see new Discussion subsection “Animals maximize rewards”).

**Reviewer #2**

2- The visual inspections of the adjustments point at a very abrupt and near immediate adjustments, which also favors representational and computational accounts (see also Tosun et al., 2016 listed above). To this end, I suggest authors to also look at the following papers:

- Kheifets, A., & Gallistel, C. R. (2012). Mice take calculated risks. Proceedings of the National Academy of Sciences of the United States of America, 109(22), 8776–8779. https://doi.org/10.1073/pnas.1205131109

- Gallistel, C. R., King, A. P., Gottlieb, D., Balci, F., Papachristos, E. B., Szalecki, M., & Carbone, K. S. (2007). Is matching innate?. Journal of the experimental analysis of behavior, 87(2), 161–199. https://doi.org/10.1901/jeab.2007.92-05

The last paper I listed is particularly relevant in consideration of the matching law that the authors mention in the paper.

**Response**

Thank you for this suggestion. We were aware of the abrupt changes described by Gallistel and others (e.g., PNAS 101: 13124), and, prompted by your request, have looked more closely at this issue.

First, please note that we plot session-by-session values (e.g., new Figure 3c), which constitute averages over several hundred trials, while Gallistel and colleagues show such abrupt changes at a trial-by-trial level, which could be masked in our plots.

To our knowledge, such abrupt adjustments have not been reported in the literature on perceptual decision making, and our experiment was not designed to arbitrate between models positing gradual or abrupt changes. We therefore applied Gallistel et al.’s (2004) changepoint detection algorithm to trial-by-trial cumulative response curves. While we did indeed observe multiple significant changepoints, these also occurred (with similar frequency) in simulated data generated by our SDT-based models. Thus, the statistical detection of changepoints does not rule out a gradual updating mechanism, at least in our data. In any case, it is not clear how a criterion learning model with abrupt criterion updates could be implemented, and the notion of whether performance changes gradually or abruptly is still debated (e.g., Forsgren et al., Psychological Review 130: 1383).

Given that our data does not allow to make any strong claims about the abruptness of change, we decided not to take up this aspect in Discussion.

**Reviewer #2**

- Finally, albeit the authors offer a "mechanistic" approach, their approach is still descriptive. A fully generative approach should account for the behaviors in their full complexity (e.g., drift diffusion model), which includes response times in the authors' work.

Overall, this is a beautiful paper, which addresses an important research question.

**Response**

Thank you for your kind words.

We agree that our model, while offering a mechanistic account of the criterion setting process, falls far short of explaining perceptual choices in their full complexity. Our models are, in principle, compatible with drift diffusion models (DDM); criterion learning rules would adjust the decision boundaries to the accumulation process, and this would produce predictions for response time distributions.

Our experiments did not encourage fast responding – animals were forced to maintain nose poking / key pecking during stimulus presentation and a short period thereafter, hampering the interpretation of reaction times (because our reaction times not only include movement times but also the animals’ ability to withhold responding for a certain amount of time). With that caveat in mind, we have analyzed reaction times to test the hypothesis that animals respond more rapidly in rich than lean conditions and found some trend in the data (p=0.086, see Discussion). Taking up the analysis of reaction times is clearly an excellent opportunity for future experiments and modeling efforts, and we are grateful for your suggestion.

We acknowledge these (and other) limitations of our model in the discussion section “Limitations and future directions” of the revised manuscript.
